# Consensus furin cleavage sites in the cuticular collagens DPY-17 and SQT-3 are required for Q neuroblast left-right asymmetric migration in *Caenorhabditis elegans*

**DOI:** 10.17912/micropub.biology.001526

**Published:** 2025-03-04

**Authors:** Vedant D. Jain, Celeste J. Gormly, Erik A. Lundquist

**Affiliations:** 1 Molecular Biosciences, University of Kansas, Lawrence, Kansas, United States

## Abstract

Previous studies showed that the apically secreted cuticular collagens DPY-17 , SQT-3 , and DPY-14 control the left-right asymmetric migration of the Q neuroblasts in *Caenorhabditis. elegans* . Furthermore, apical secretion of DPY-17 and SQT-3 require the BLI-4 proprotein convertase of the subtilisin/kexin family and the consensus furin cleavage site (CFCS) in the N-terminus of DPY-17 and SQT-3 . Work here shows that the CFCS sites of DPY-17 and SQT-3 are required for their roles in Q neuroblast migration. *bli-4* mutants had only weak effects on Q neuroblast migration, possibly due to redundancy among isoforms. These results suggest that apical secretion of cuticular collagens is required for Q neuroblast migration. These collagens might themselves provide left-right asymmetric guidance information, or might regulate another aspect of Q cell interaction with the cuticle, such as adhesion.

**Figure 1. AQR and PQR migration defects in dpy-17, sqt-3, dpy-14, and bli-4 mutants:**
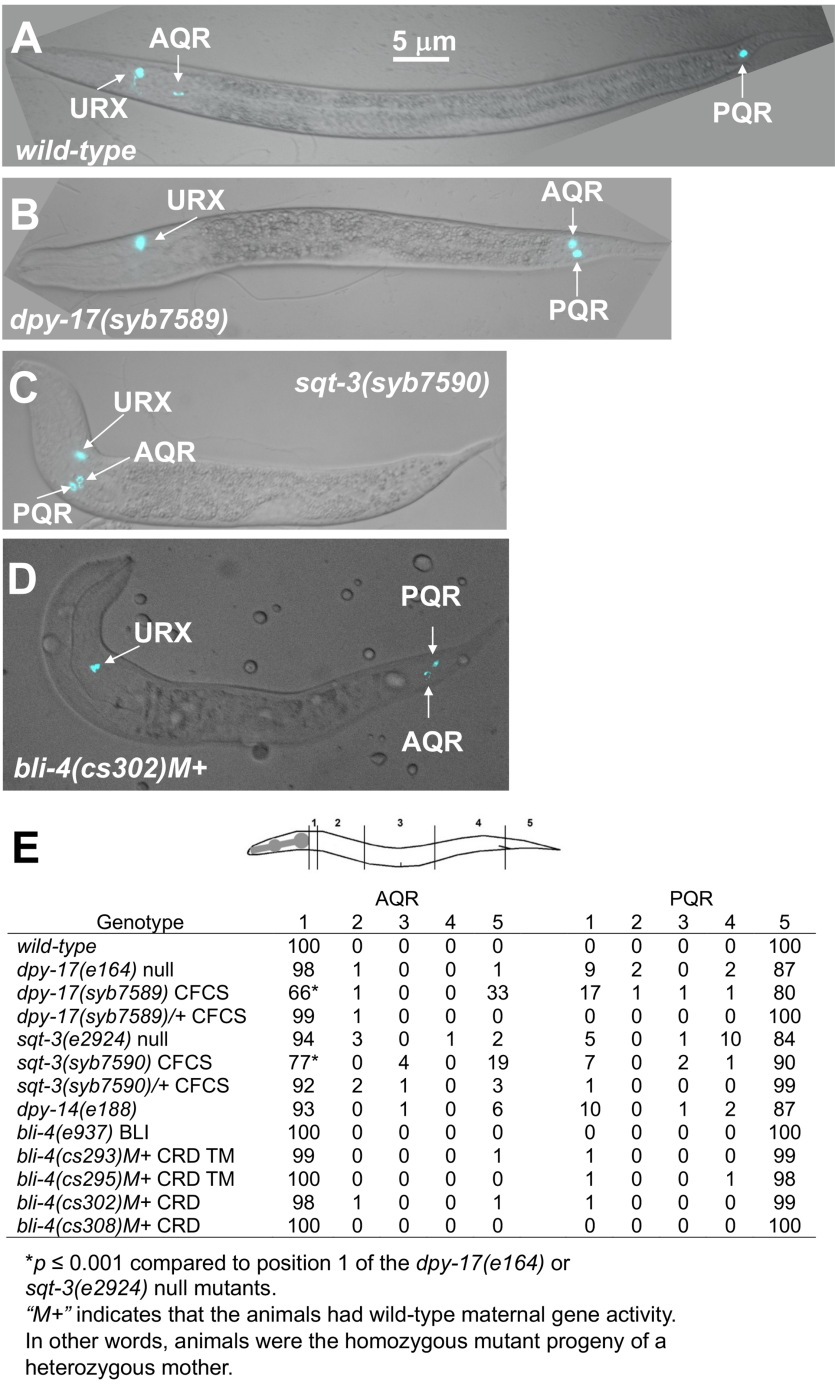
A-D) Fluorescent and DIC merged images of animals with *Pgcy-32::cfp* expression (cyan). The URX, AQR, and PQR neurons are indicated. The scale bar in A represents 5 μm. Genotype names of *dpy-17(syb7589)* and *sqt-3(syb7590)* were shortened for space and do not include the *dpy-17(syb3685)* and *sqt-3(syb3691)* gfp insertions also in the strains. Images were acquired in the *cfp* fluorescence channel, so GFP from the gene tags is not visible in the figures. M+ indicates that the animal had *wild-type* maternal gene activity (i.e. was a homozygous mutant progeny of a heterozygous mother). A) In *wild-type* , AQR is in the head deirid ganglion (position 1), and PQR is in the phasmid ganglion posterior to the anus (position 5). B) Both AQR and PQR migrated posteriorly to the phasmid ganglion in a *dpy-17(syb7589)* mutant. C) Both AQR and PQR migrated anteriorly to the deirid ganglion in a *sqt-3(syb7590)* mutant. D) Both AQR and PQR migrated posteriorly in a *bli-4(cs302)* mutant. E) A table showing the positions of AQR and PQR in different genotypes. For each genotype, 100 animals were scored. *dpy-17(e164)* and *sqt-3(e2924)* data taken from (Lang and Lundquist 2021). The asterisk (*) represents *p* ≤ 0.001 compared to the null allele at that position (Fisher's exact test). The diagram above the table represents the five positions along the anterior-posterior axis of the animal. Q neuroblasts are born in position 4. CFCS indicates a consensus furin cleavage site mutant. CRD represents a cysteine rich domain containing isoform mutant. CRD TM represents a mutant in the isoforms containing the cysteine-rich domain and transmembrane domain. BLI indicates a mutation in the viable Bli phenotype isoforms.

## Description

The bilateral Q neuroblasts, sisters of the V5 seam cells are born during embryogenesis and migrate in the L1 larva (Sulston 1976; Chapman *et al.* 2008; Middelkoop and Korswagen 2014). On the right side, QR migrates anteriorly over the V4 seam cell, and on the left side, QL migrates posteriorly over the V5 seam cell (Chapman *et al.* 2008). An identical pattern of cell division, cell death, and neuronal differentiation produces three neurons on each side: AQR, AVM, and SDQR on the right, and PQR, PVM, and SDQL on the left. QR descendants migrate anteriorly. AQR migrates the farthest to the deirid ganglion near the pharynx ( Figure 1 ). QL descendants migrate posteriorly. PQR migrating the farthest posteriorly to the phasmid ganglion posterior to the anus ( Figure 1 ).

The transmembrane receptor molecules UNC-40 /DCC, PTP-3 /LAR, MIG-21 , CDH-3 and CDH-4 control initial Q migration (Middelkoop *et al.* 2012; Sundararajan and Lundquist 2012; Sundararajan *et al.* 2014; Ebbing *et al.* 2019). UNC-40 /DCC and PTP-3 /LAR act redundantly in parallel in QL to promote posterior migration, and mutually inhibit each other's activity in QR, causing anterior migration. Initial migration defects cause subsequent errors in migration of AQR and PQR (Chapman *et al.* 2008).

Mutations in the cuticle collagen genes *dpy-14 , dpy-17* , and *sqt-3* cause initial Q migration defects similar to *unc-40* and *ptp-3* , as evidenced by reversal of AQR and PQR migration (Lang and Lundquist 2021; Lundquist 2024) . AQR is sometimes in the normal posterior position of PQR, and PQR is sometimes in the normal anterior position of AQR ( Figure 1 ) *.* DPY-17 and SQT-3 likely act together in a collagen trimer and are mutually required for each other secretion (Novelli *et al.* 2006; Birnbaum *et al.* 2023). AQR/PQR migration defects in double mutants of each combination resemble defects in single mutants, suggesting that they all act in a common pathway, possibly in a DPY-14 / DPY-17 / SQT-3 collagen trimer (Lang and Lundquist 2021; Lundquist 2024) . The proprotein convertase of the subtilisin/kexin family (PCSK) BLI-4 is required for proper apical secretion of DPY-17 and SQT-3 to form the cuticle (Birnbaum *et al.* 2023). Cleavage of DPY-17 and SQT-3 at an N-terminal dibasic consensus furin cleavage site (CFCS) by BLI-4 is required for secretion (Birnbaum *et al.* 2023). Mutations in *bli-4* cause improper DPY-17 and SQT-3 secretion and cuticle defects. Mutations of the CFCS sites in *dpy-17* and *sqt-3* cause similar defects (Birnbaum *et al.* 2023). DPY-14 also has a predicted N-terminal CFCS site (…STAGKSGY *R* AK *R* AWQFGSWV…) but its function has not been investigated.

Work described here shows that the CFCS sites of DPY-17 and SQT-3 are required for Q neuroblast migration. *dpy-17 ( syb7589 )* and *sqt-3 ( syb7590 )* CFCS mutants displayed AQR and PQR migration defects that resembled null alleles, with AQR sometimes in the tail and PQR sometimes in the head ( Figure 1 ). The penetrance of PQR defects of CFCS mutants were not significantly different than nulls ( Figure 1 ). However, AQR defects were significantly stronger in the CFCS mutants. This suggests that the CFCS mutants could have a dominant interfering effect on other factors necessary for AQR migration, possibly by interfering with their secretion ( *e.g.* another unidentified cuticle collagen that might be involved). Indeed, *sqt-3 ( syb7590 )/+* heterozygotes displayed dominant Dpy and right-hand roller phenotypes, and dominant AQR and PQR migration defects ( Figure 1 ). *dpy-17 ( syb7589 )/+* heterozygotes were weakly Dpy but did not display AQR/PQR migration defects. These results indicate that the *sqt-3 ( syb7590 )* mutant is dominant and might have dominant-negative effects on cuticle formation and on AQR/PQR migration. DPY-17 and SQT-3 are mutually required for each other's secretion (Novelli *et al.* 2006; Birnbaum *et al.* 2023). In *dpy-17* mutants, much SQT-3 remains in the cytoplasm in a pattern consistent with the endoplasmic reticulum (Birnbaum *et al.* 2023). In *sqt-3* mutants, DPY-17 protein level is severely reduced (Birnbaum *et al.* 2023). This suggests that SQT-3 is required for the stability of DPY-17 and possibly other proteins, and might explain the dominant-negative effect of *sqt-3 ( syb7590 )* but not *dpy-17 ( syb7589 )* .

The classic *bli-4 ( e937 )* viable blistered cuticle mutant had no defects in AQR or PQR migration ( Figure 1 ). *bli-4* encodes at least four groups of isoforms, with viable *bli-4 ( e937 )* affecting only the BLI isoform group (Birnbaum *et al.* 2023). Mutations predicted to affect all isoforms ( *i.e.* *bli-4* nulls such as *bli-4 ( cs281* )) are embryonic lethal and could not be scored for AQR/PQR migration, which occurs in the L1 larva. Mutations that specifically affect the cysteine-rich domain (CRD) isoforms with and without a transmembrane domain (TM) result in larvally-arrested animals with defective cuticles (Birnbaum *et al.* 2023). These mutants had variable defects in AQR and PQR migration (0-2%), less penetrant than *dpy-17* and *sqt-3* mutants ( Figure 1 ). Possibly, different isoforms of BLI-4 can act with redundancy on DPY-17 and SQT-3 CFCS cleavage. The astacin metalloprotease DPY-31 is thought to process SQT-3 at a C-terminal site (Novelli *et al.* 2004; Novelli *et al.* 2006), but *dpy-31* mutants had no effect on AQR/PQR migration (Lang and Lundquist 2021) . This is consistent with other results that show that some aspects of SQT-3 function do not require DPY-31 (Novelli *et al.* 2004; Novelli *et al.* 2006; Birnbaum *et al.* 2023).

These results indicate that factors affecting cleavage and apical secretion of DPY-17 and SQT-3 to form the apical extracellular matrix cuticle are required for their roles in Q neuroblast migration. It is unclear how the cuticle might be providing left-right asymmetric guidance information to the Q neuroblasts. Possibly, cuticle structure affects the structure of the underlying basal extracellular matrix basement membrane upon which the Q neuroblasts migrate. EPI-1 /lamininA is required for the ability of the Q descendants to migrate but does not affect direction (Lang and Lundquist 2021) . EMB-9 /Collagen IV A1 mutants have low penetrance PQR directional migration defects (0-2%) (Lang and Lundquist 2021) , suggesting a possibly role of basement membrane collagen IV in QL direction. The Q cells are the daughters of the lateral epidermal seam cells and are in contact with the cuticle when born before they migrate between the basement membrane and epidermis (Sulston and Horvitz 1977; Chapman *et al.* 2008; Middelkoop and Korswagen 2014). Possibly, DPY-14 , DPY-17 , and SQT-3 in the cuticle provide the left-right asymmetric guidance information required for initial Q neuroblast migration. Alternately, DPY-14 , DPY-17 , and SQT-3 might affect Q cell attachment to the cuticle, which might influence migration. This is consistent with DPY-17 acting with MUA-3 /Fibrillin1 in muscle cell and organ attachment to the cuticle. In any case, these data indicate that collagens in an apical extracellular matrix can influence neuroblast migration.

## Methods

Standard *C. elegans* culture and genetic techniques at 20°C were utilized (Brenner 1974; Fay 2013) . A *Pgcy-32::cfp* transgene ( *lqIs244 )* was used to visualize AQR and PQR (Chapman *et al.* 2008; Josephson *et al.* 2016). A five-position scale was used to assess AQR and PQR position along the body as previously described (Josephson *et al.* 2016) (see diagram in Figure 1E ): position 1-the normal position of AQR in the deirid ganglion in the anterior; position 2-posterior to the normal position of AQR and anterior to the vulva; position 3 adjacent to the vulva; position 4-the birthplace of the Q neuroblasts in the posterior; and position 5-the normal final position of PQR in the tail in the phasmid ganglion behind the anus. Significance of difference of proportional data between genotypes was determined using Fisher's Exact test. Wormbase (Sternberg et al., 2024) was used for *C. elegans* informatics.

## Reagents

The following *C. elegans* strains and genotypes were utilized:

**Table:** 

Strain	Genotype	Origin
N2	*wild-type*	CGC
LE3885	*lqIs244 II[Pgcy-32::cfp] *	Josephson et al., *2016*
LE4752	*dpy-17 ( e164 ) III; lqIs244 II *	Lang and Lundquist, 2021
LE7646	*dpy-17 ( syb7589 syb3685 ) III; lqIs244 II *	This work/Birnbaum et al., 2023
LE4761	*sqt-3 ( e2924 ) V; lqIs244 II *	Lang and Lundquist, 2021
LE7625	*sqt-3 ( syb7590 syb3691 ) I; lqIs244 II *	This work/Birnbaum et al., 2023
LE6871	*dpy-14 ( e188 ) I; lqIs244 II *	Lang and Lundquist, 2021
LE6785	*bli-4 ( e937 ) I; lqIs244 II *	This work/CGC
LE7697	*bli-4 ( cs293 ) I; lqIs244 II; csEx919 [ bli-4 (+)] *	This work/Birnbaum et al., 2023
LE7671	*bli-4 ( cs295 ); lqIs244 II; csEx919 [ bli-4 (+)] *	This work/Birnbaum et al., 2023
LE7699	*bli-4 ( cs302 ); lqIs244 II; csEx919 [ bli-4 (+)] *	This work/Birnbaum et al., 2023
LE7696	*bli-4 ( cs308 )/ szT1 I; lqIs244 II *	This work/Birnbaum et al. *,* 2023
LE7695	*bli-4 ( cs281 ) I; lqIs244 II; csEx919 [ bli-4 (+)] *	This work/Birnbaum et al., 2023
